# Static test research on steel bridge deck pavement structures paved by high-content hybrid fibre polymer concrete

**DOI:** 10.1038/s41598-022-12987-8

**Published:** 2022-05-25

**Authors:** Chaohua Zhao, Zhiwei Zhu, Zhijian Yi, Kang Su, Ya Li

**Affiliations:** 1 School of Civil Engineering, Chongqing Jiaotong University, Chongqing, 400074 China; 2 Institute of Scientific Innovation, China Merchants Chongqing Highway Engineering Testing Center Co., Ltd, Chongqing, 400060 China

**Keywords:** Engineering, Materials science, Mathematics and computing

## Abstract

Steel bridge deck pavement has always been a key and difficult point in the construction of long-span bridges. In practical engineering, common paving material is asphalt, and serious damage is caused on the pavement layer in the early stage. In this study, a high-performance high-content hybrid fibre polymer concrete was used as the paving material. A test was conducted on the small beam of the composite structure formed by the pavement layer and steel plate, and a positive/negative bending moment test was conducted to analyse the stiffness and bearing capacity of the composite structure. As revealed in the research results, the flexural and tensile stiffness of the structure and the bearing capacity of the composite structure showed superior performance, increased significantly with the pavement thickness, but increased slowly after the pavement thickness exceeded 80 mm and the increase in thickness contributed little to the bearing capacity. Under the simulated action of a positive/negative bending moment, the pavement layer still exhibited certain ductile failure features when the structure was bearing an ultimate load. This proves that high-content hybrid fibre polymer concrete exhibits suitable mechanical properties for steel bridge deck pavement.

## Introduction

Steel bridge deck pavements remain a key and challenge in the construction of long-span bridges. In actual engineering, serious defects occur on pavement layers in an early stage^1^. Cement concrete is a common pavement material but is rarely used for steel bridge deck pavements because it is brittle, with a small allowable limit deformation. When ordinary cement concrete is utilised in the pavement of ordinary cement concrete roadbeds without reinforcement, many vertical and horizontal cracks appear on the surface or inside the concrete, which can be detected by visual inspection or by more advanced non-destructive testing methods^2–6^. Due to the high flexibility of orthotropic steel bridge decks, a large difference between pavement layer deformation and bridge decks is obtained under vehicle loading; thus, traditional cement concrete cannot be applied to steel bridge decks. Currently, asphalt is maturely and commonly used for steel bridge deck pavement.

**Figure 1:**
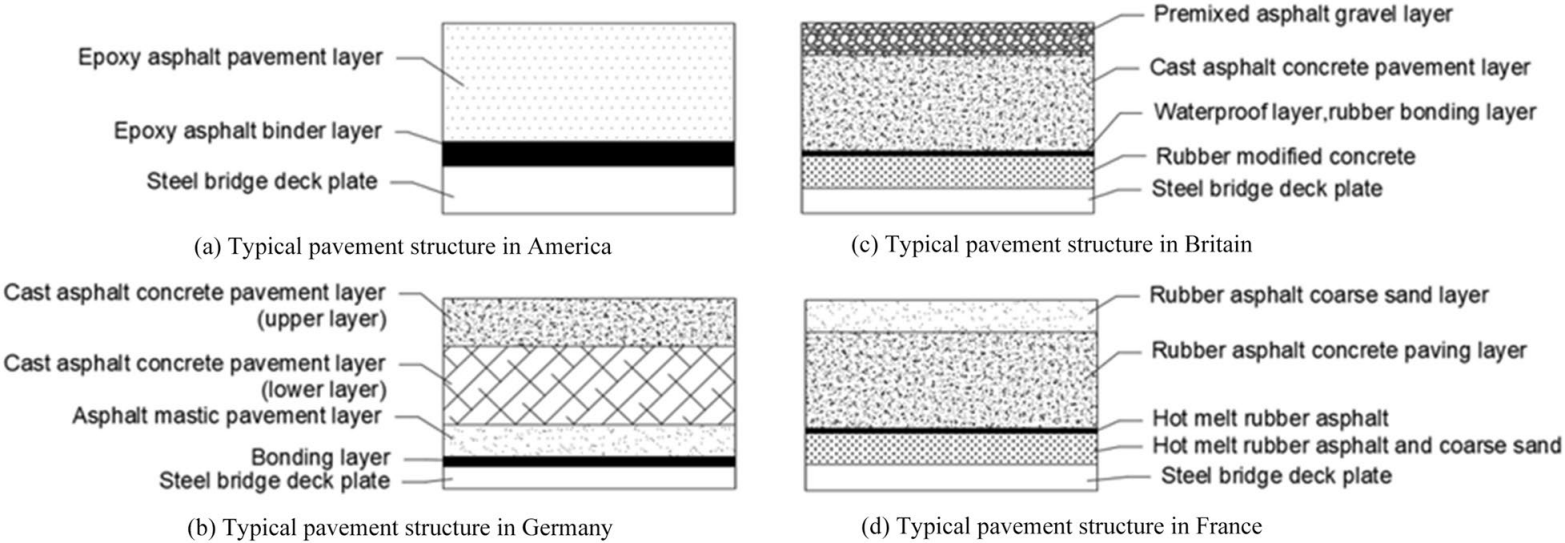
Typical pavement structures of steel bridge deck. (**a**) Typical pavement structure in America; (**b**) typical pavement structure in Germany; (**c**) typical pavement structure in Britain; (**d**) typical pavement structure in France.

Steel bridge deck pavement structures are generally divided into single- and multilayer pavement systems. Different countries or regions have different climates (e.g., extreme temperature and rainfall) and traffic volumes; thus, the pavement scheme must be selected on the basis of the specific engineering experience. In America, most steel bridge decks are paved using single-layer epoxy asphalt. This technology is relatively mature^7–9^. The structural form is as presented in Fig. 1a. In Germany, the pavement of a steel bridge deck is complicated, with multiple layers and high pavement thickness. The structural form is displayed in Fig. 1b. In Britain, a typical steel bridge deck has many pavement layers, but the total thickness is merely approximately 4 cm. The structural form is illustrated in Fig. 1c. In France, a typical steel bridge deck has four pavement layers, with a total thickness of approximately 5 cm. The structural form is illustrated in Fig. 1d.

However, the asphalt may exhibit many issues that occur in practical engineering. For example, hot mixed asphalt concrete needs a high environment and mixing temperatures, which restrict the construction time, resulting in a short construction period during the entire year^10–12^. Grouted asphalt concrete^6,10,13–15^ exhibit low durability when used as the top layer of steel bridge deck pavement^15–17^. Modified asphalt concrete needs a large thickness when used for steel bridge deck pavement^18,19^. Longitudinal cracks, pit slots, or interlayer voids can often be observed on pavement layers^20,21^ when using epoxy asphalt^22–24^ as steel bridge deck pavement.

Based on the above-mentioned analysis, it can be easily observed that conventional asphalt shows many disadvantages which are detrimental to the long-term service performance of bridge decks. Therefore, more and more researchers are focusing on improving the mechanical properties of concrete. The ultrahigh-performance concrete (UHPC) and polyurethane concrete are proposed and widely used in the construction of steel bridge decks accordingly in very recent decades. For example, in 2002, UHPC was used to pave the sidewalk slab of a bridge on the Qinghai-Tibet Railway of China, which presents harsh environmental conditions. Through a study on the steel–UHPC lightweight composite structure, Xudong^26–28^ proved that with a certain dead weight, this structure can improve the stiffness of the pavement structure and reduce the stress on the pavement layer. This structure has been then applied to Beijiang Bridge No. 4 in Qingyuan City, Guangdong Province, and the Dongting Lake Bridge. As for the polyurethane concrete, the concrete was first used to pave a steel bridge deck for the channel of the South Hongmei Road in Shanghai, China^29^ in 2017. It is well known that the concrete is a brittle material^30^, the material is not tension resistant and cannot resist impact and cyclic loads. The technique of adding fibres (e.g. steel fibres) to concrete is also considered to be a more effective method of improving the properties of concrete, such as shear, flexural and tensile properties^31–34^. To date, although there has been researched into low-mix polymer concrete, the concrete materials available still cannot meet the requirements for crack resistance and toughness of road materials. That is, none of the existing studies has reported high fibre admixtures for polymeric concrete, with a typical fibre admixture of no more than 3% by volume 34, 41. To further improve cracking resistance and achieve ultra-high toughness, a series of high-performance polymer concretes with high strength, toughness, and environmental adaptability has been studied and realised^35–37^ by the authors. As reported in the literature^37^, hybrid fibre polymer concrete exhibits reliable working performance, with the elastic modulus, compressive strength, and flexural strength reaching up to 35.93 GPa, 52.82 MPa, and 11.51 MPa, respectively. Based on the authors’ engineering practice, the cost of the pavement structure proposed through the technical modifications by the authors does not exceed 800 RMB/m^2^, with cost savings of 50% at least. Moreover, it exhibits high sulfate erosion resistance, which makes it suitable for pavement under special environmental conditions.

As can be seen in the above literature review, the investigations on high-performance polymer concrete generate great methods for improving the properties of the material. However, none of the previous investigations has performed the structural analysis on such high-performance polymer hybrid fibre concrete. The lack of structural studies of the developed materials may limit the application of the materials in practical engineering. Meanwhile, the lack of research into mechanical mechanisms also weakens the scientific character of the materials developed by the authors and is not conducive to widespread application. To further illustrate the superiority of high fibre admixture polymer concrete (Fibres are mixed at 5% or even up to 7% by volume) and enhance the research on mechanical mechanisms of the concrete, this paper aims to chose the optimum proportion of materials for the structural tests to obtain the performance of the current polymer hybrid fibre concrete. Meanwhile, a strong and durable bonding system is also introduced in this paper for steel bridge deck pavement structures.

## Materials and test method

### Test purpose and design

This paper discusses two pavement structures: a single-layer pavement structure (a high-content hybrid fibre polymer concrete pavement layer) and a double-layer pavement structure (where the upper layer is a polymer lattice concrete wear layer and the lower layer is a high-content hybrid fibre polymer concrete pavement layer). The two structures are introduced as follows.

#### Single-layer pavement structure

**Figure 2:**
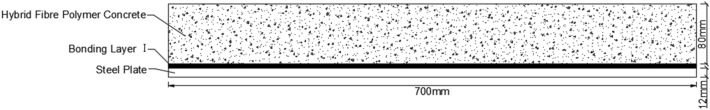
Single-layer pavement structure.

The pavement layer had an area of 700 mm (L) × 100 mm (W) and a thickness of 60/70/80/90 mm. Each group comprised three test pieces (to reduce error, the average of the three test results was taken; if one of the results was more than 15% different from the other two results, the result was ignored). In addition, the thickness of the interface bonding layer was ignored. The dimensions of the steel plate were 700 mm × 100 mm × 12 mm, and this plate was bonded to the pavement layer through modified epoxy resin. The single-layer pavement structure is presented in Fig. 2, where t = 60–90 mm.

#### Double-layer pavement structure

**Figure 3:**
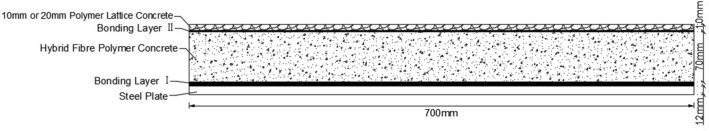
Double-layer pavement structure.

For the double-layer pavement structure, the lower layer was a 70-mm-thick high-content hybrid fibre polymer concrete pavement layer, and the upper layer was a 10- or 20-mm-thick wear layer paved using polymer lattice concrete; the two layers were bonded with bonding layer II. The specific test steps were similar to those followed for the single-layer pavement structure. Before grouting the wear layer, the lower layer was cured for > 14 days. The polymer lattice porous concrete for grouting the wear layer was made compact by using a special tool. The double-layer pavement structure is illustrated in Fig. 3.

For the static performance of the structure, the following aspects are mainly studied in this paper:

1. The mechanical properties and failure modes of single-layer pavement structures with thicknesses of 60, 70, 80, and 90 mm;
2. The mechanical properties and failure modes of double-layer pavement structures with thicknesses of 10 mm + 70 mm and 20 mm + 70 mm;
3. The mechanical properties and failure modes of the two pavement structures under the action of positive and negative bending moments.

Analytical contents included the flexural and tensile stiffness of the members, which were mainly reflected through the load–displacement curve of the structure, as well as the failure modes of the members, the appearance and expansion of cracking, and the influence of the pavement structure on enhancing the flexural and tensile stiffness of steel plates.

### Raw materials

Cement: ordinary Portland cement with nameplate of ‘Huaxin Cement’ and label of P.O42.5 produced in Chongqing;

Coarse aggregate: Granite gravels with size grading of 5–10 mm and maximum nominal particle size of 10 mm produced in Chaoyang River, Chongqing;

Fine aggregate: medium-coarse sand produced in Chongqing;

Fly ash: First-grade fly ash produced by Luohuang Power Plant in Chongqing;

Ordinary steel fibre: Corrugated shear-type ordinary steel fibre produced by Anshan Kebite Technology Development Co., Ltd.;

Ultrashort ultrafine steel fibre: Melt-drawing ultrashort ultrafine steel fibre produced by Anshan Kebite Technology Development Co., Ltd.;

Flexible fibre: Polypropylene flexible fibre produced by Langfang Haoxin Thermal Insulation and Fireproof Sealing Material Co., Ltd. in Shandong;

Emulsion: Styrene butadiene rubber (SBR)-modified polymer latex.

Table 1 presents the main physical and mechanical parameters of three different fibres: ordinary, ultrashort ultrafine, and flexible steel fibres. It should be noted that the formula presented in Table 1 is the optimum fibre formulation. More details about the choice of the current formulation can be seen in the authors’ previous research^37,38^. Under the proposed fibre combination, this paper conducted the following structural analysis.

**Table 1:** Main physical and mechanical properties of the three types of fibres.

Fiber type	Length, in mm	Diameter, in mm	L/D	Bending performance	Elasticity modulus, in GPa	Tensile strength, in MPa
Ordinary steel fiber	35	0.87	40	> 90	210	425
Ultrashort ultrafine steel fiber	6	0.2	30	> 90	240	880
Flexible fiber	12	0.03	400	> 90	3.85	500

Table 2 presents the mass mixture ratio in each cubic meter of hybrid fiber polymer concrete for the pavement structure layer.

**Table 2:** Masses of materials per cubic volume.

Type	Cement	Gravel	Sand	Ordinary steel fiber	Ultrashort ultrafine steel fiber	Flexible fiber	Polymer emulsion	Fly ash
Mass per volume, in KG	580	600	600	150	312	3	320	250

Among them, the volume rates of ordinary steel fibres and ultrashort ultrafine steel fibres were 1.91% and 4%, respectively, and the polymer-cement ratio was 0.55.

The polymer-modified cement lattice concrete for paving the wear layer is a mixture of 5–10 mm discontinuously graded broken granite aggregates, 42.5 ordinary Portland cement, and polymer emulsion in the ratio of 1600:400:120 kg by mass per unit cubic volume. The discontinuously graded broken stones that formed the lattice structure inside the concrete provided the concrete strong anti-shrinking and deformation coordination ability and thus improved the ease of formation. Moreover, through bonding, this layer can be well integrated with the lower layer of high-content hybrid fibre polymer concrete.

### Preparation of paving materials

The high-content hybrid fibre polymer concrete for paving the structural layer was mixed and produced with reference to the preparation method of relevant polymer steel fibre concrete^39^, and the steps followed for preparing the high-content hybrid fibre polymer concrete used in this study are as follows:

1. The two fibres were poured out in different positions and dispersed separately; the corroded and clustered steel fibres were removed.
2. Sand and broken stones were oven-dried at 105 °C without any decrease in weight and then removed.
3. The weighed coarse and fine aggregates and cement were placed in a mixing pot and mixed for approximately 1 min; then, the two fibres (steel fibre and flexible fibre) were gradually added to the mixture and mixed for approximately 1 min again to facilitate their even dispersion in the aggregates.
4. The polymer emulsion was slowly added into the mixture and stirred.
5. The final mixture was discharged to prepare the test piece. In preparation, the mixture was vibrated to shape up. The vibrating time in the vibrating table was kept neither considerably short nor long to ensure compactness, prevent the steel fibres from sinking, and avoid the uneven distribution of the density of each layer of concrete.

The polymer lattice porous concrete for paving the wear layer was prepared in a manner similar to the mixing preparation of ordinary cement concrete, as described in the literature^35,36^.

### Preparation of pavement structure test pieces

Test steps:

1. A wooden mold with the corresponding size was prepared; the steel plate was polished on a grinding machine; then, the flatness of the steel plate was tested, and the steel plate was put into the wooden mold with the rust-removed surface facing upwards.
2. The surface was brushed with an interface-bonding agent at a spreading rate of approximately 1 kg/m^2^ to ensure that the steel plate surface was evenly painted.
3. High-content hybrid fibre polymer concrete was poured onto the bonding interface and vibrated to shape up; three days later, the wooden mould was removed.

For double-layer pavement, the wear layer was poured into shape the next day after the first layer of hybrid fibre polymer concrete solidified. The interface-bonding agent was also brushed on the wear layer after the wear layer shaped up.

Curing method: the pavement layer of the material was cured in a wetting manner first and then in a drying manner. The layer was watered and covered with a film for one day of wet curing; then, the mould was removed, and the pavement layer was watered and filmed again for two days of curing and then cured in a dry environment at ambient temperature. After curing for 28 days, test pieces were subjected to a loading test.

### Loading method

In the loading test, the third-point loading scheme of ordinary anti-flection concrete test pieces was used for reference^40^. In this scheme, the space between the supports was 600 mm, and those between the loading points and between the support and loading point were both 200 mm. The test piece was wiped with a rag. The centre below the test piece was pasted with a strain gauge, and the centre of the test piece was mounted with a dial indicator; wood chips were pasted on two sides of the test piece, which were supported by a dial indicator header. The dial indicator and strain gauge were both connected to a YE2539 high-speed static strain indicator under normal conditions, and strain gauges were pasted on the same batch of test pieces for temperature compensation. The press, with a power of 200 kN, was turned on, followed by start-up of the oil pump, slow adjustment of the oil delivery and return valves until the oil pressure reached the normal level; after that, the two valves were closed. After the wiring was well connected, the test piece was raised to a suitable height and carefully adjusted to closely contact the press header; then, the oil delivery valve was rotated slowly to increase the load evenly and slowly. While the load was read, the dial indicator collected the displacement and scanning strain value continuously. When failure finally appeared on the test piece, the test was ended. The test results and phenomenon were recorded.

Compared to the third-point loading of ordinary cement concrete, the loading of the pavement structure test piece had the following differences:

(1) The sizes of the test pieces were different.

The flexural test piece of ordinary cement concrete was only 400 mm in length, while that of the steel bridge pavement structure in this study was 700 mm. The calculated span between the supports was 600 mm, and the third point (loading point) from the support was 200 mm.

(2) A strain gauge was provided to test the strain of the structural test pieces, while a dial indicator was added to test the deflection of the structural test pieces.

In the literature y^40^, the purpose of loading on the flexural test piece of ordinary cement concrete was to measure the flexural tensile strength of the material, regardless of the deformation and deflection. For the test piece of the pavement structure, the deflection of the structural test piece and the strain of the material should be examined considering the test purpose. Therefore, a strain gauge should be provided to test the change in the strain in the tensile material at the bottom of the loaded test piece, and a displacement dial indicator should be added to test the deflection displacement of the structural test piece with different loads.

(3) The loading points of the test pieces were on different planes.

When the flexural test piece of ordinary concrete was loaded, the side that was going to shaped up faced upwards to ensure the flatness of the support and loading planes and to reduce error. When the test piece of the pavement structure was shaped up, the steel plate was at the bottom of the test mould. However, in actual engineering, the tensile conditions of the pavement layer and the steel plate should be considered due to the specificity of steel bridge deck pavement (the tension is on the upper side).

To test the flexural and tensile strain of the steel plate or pavement layer in the hybrid fibre polymer concrete pavement structure, two loading methods according to the direction are available: positive bending moment simulation loading and negative bending moment simulation loading.

**Figure 4:**
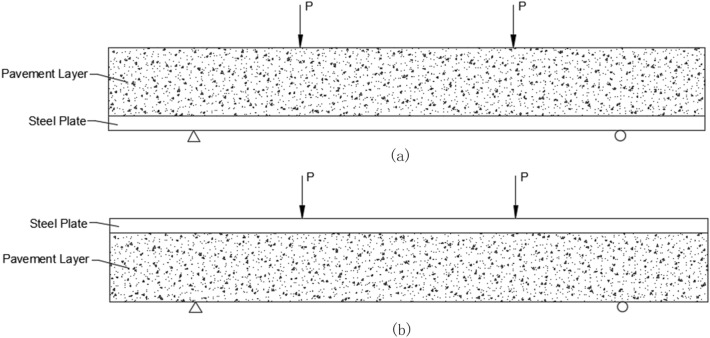
Schematic of loading for test. (**a**) Schematic of positive bending moment loading; (**b**) schematic of negative bending moment loading.

Positive bending moment simulation loading: In this method, the bottom of the steel plate is on the lower side, while the pavement layer is on the upper side and used as the loading contact surface. This method is used to simulate the pressure on the pavement layer at the positive bending moment bearing position in the actual pavement structure (Fig. 4a).

Negative bending moment simulation loading: In this method, the pavement layer is on the lower side, while the bottom of the steel plate is on the upper side and used as the loading contact surface. This method is used for simulating the tension on the pavement layer at the negative bending moment bearing position in the actual pavement structure (Fig. 4b).

## Results and discussion

### Static test on single-layer pavement structure

The entire single-layer pavement structure formed by the steel plate and a pavement layer with different thicknesses exhibit different stiffnesses. The structural stiffness under the action of a positive/negative bending moment can be expressed by the load–displacement curve of the structure.

#### Performance analysis of the pavement structure under the simulated action of positive bending moment

The flexural and tensile stiffness of the single-layer pavement structure can be measured using the corresponding load–displacement curve. Table 3 shows the load–displacement curve of the single-layer pavement structure with different pavement thicknesses (with the steel plate on the lower side) under the simulated loading of a positive bending moment. The stiffness of the composite structure is affected by the thickness of the pavement layer, and the full-process load–displacement curve under tactual flexural and tensile conditions of the structure cannot be accurately plotted. To measure the structure stiffness more accurately, it was calculated using the load–displacement curve of the member in the linear elastic stage. The flexural and tensile stiffness and failure load of the composite pavement structure formed by different thicknesses of single-layer pavement under the action of a positive bending moment are presented in Table 3.

**Table 3:** Flexural and tensile stiffness and failure load of the composite structure formed by different thicknesses of single-layer pavement.

Pavement thickness	60 mm	70 mm	80 mm	90 mm
Flexural and tensile stiffness of the composite structure, in KN/mm	12.303	17.445	33.705	38.326
Failure load, in KN	47	71	76	87

**Figure 5:**
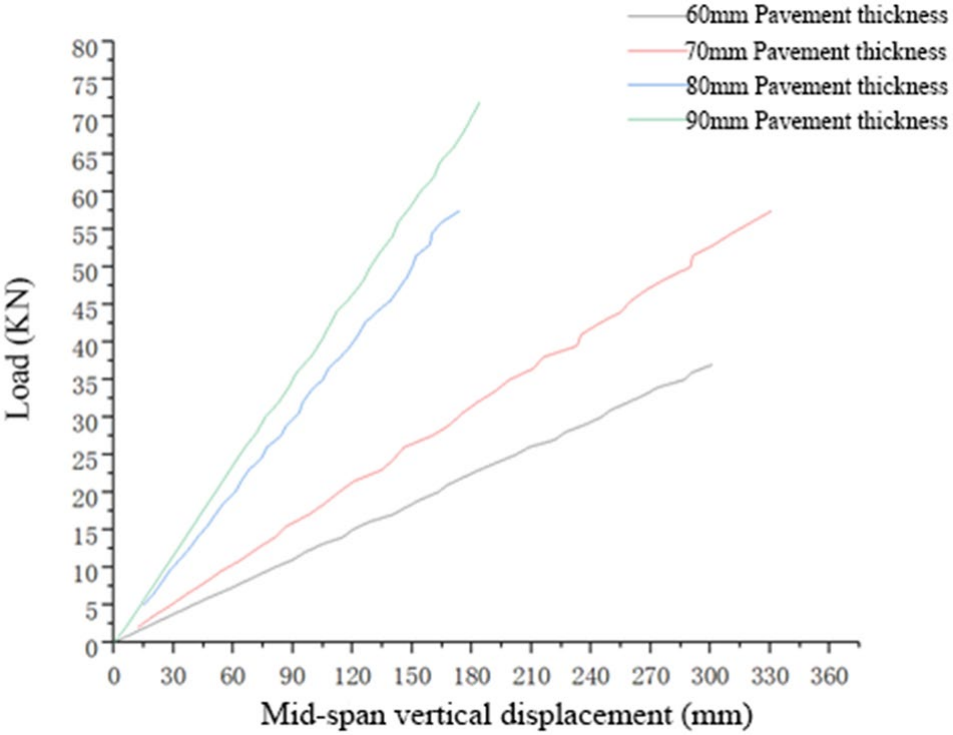
Load–displacement curve of a single-layer pavement structure with different pavement thicknesses in the linear elastic stage under the simulated action of a positive bending moment.

According to Fig. 5, the following results were obtained:

1. The flexural and tensile stiffness of the composite structure formed by the pavement layer, bonding layer, and steel plate was significantly affected by the pavement thickness. The flexural and tensile stiffness of the structure can be expressed by a load–displacement curve. According to the curve, the pavement thickness is directly proportional to the flexural and tensile stiffness of the pavement structure.

The thickness of the single–layer pavement also significantly influenced the bearing capacity of the composite structure. The thicker the pavement layer, the larger the bearing capacity of the pavement structure. The average failure load on the composite structure formed by 60-mm-thick single-layer pavement was much lower than that on the structure with the other three pavement thicknesses.

Steel fibres and flexible fibres exhibit anti-cracking enhancement effects. When the load on the test piece was increased almost to the peak, a clear "pa-pa" voice-like fibres were pulled out or broken. At this moment, the fibres were partially pulled and sheared in place of the concrete. When the crack occurred, the load continued to increase until the crack pointed near the loading cushion block. Finally, the failure on the pavement layer was shear-compression failure.

#### Performance analysis of the pavement structure under the simulated action of a negative bending moment

Under the simulated action of a positive bending moment, the force on the pavement layer was conservative. However, in practice, a negative bending moment appears on the steel bridge deck pavement above the longitudinal ribs and longitudinal and transverse partitions under the action of vehicle loading. In addition, for the pavement layer, a negative bending moment is unfavorable and thus should be the focus.

**Figure 6:**
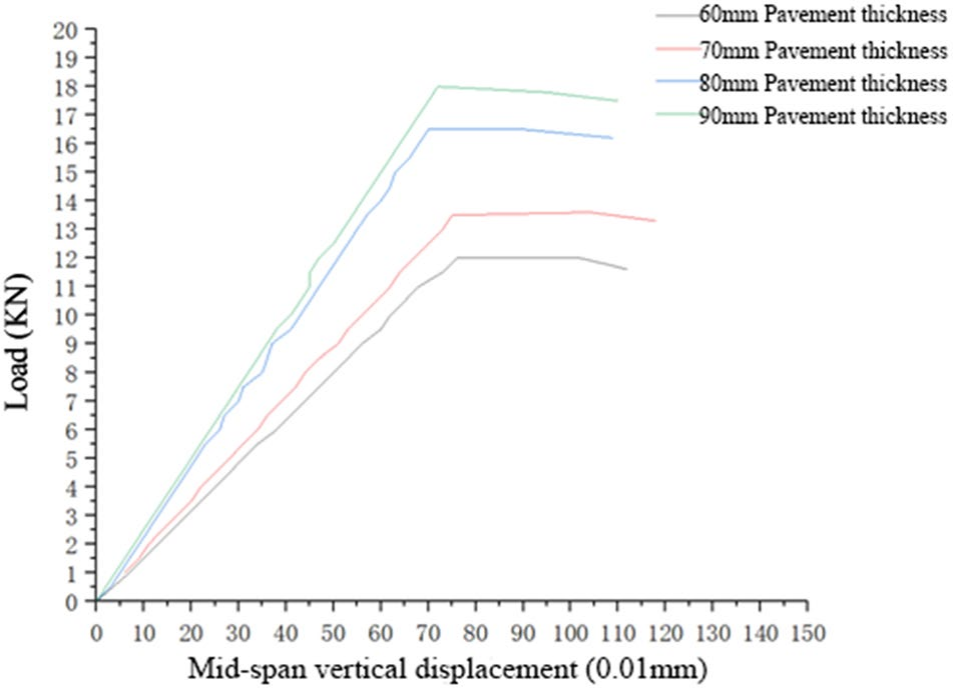
Load–displacement curve of a single-layer pavement structure with different pavement thicknesses under the simulated action of a negative bending moment.

Similar to the force analysis conducted on a positive bending moment, it is rational to select the load–displacement curve of the pavement structure under loading action in the linear elastic stage (Fig. 6). The flexural and tensile stiffness and failure load of the composite structure formed by different thicknesses of single-layer pavement under the action of a negative bending moment are illustrated in Table 4.

**Table 4:** Flexural and tensile stiffness and failure load of the composite structure formed by different thicknesses of single-layer pavement under the action of a negative bending moment.

Pavement thickness	60 mm	70 mm	80 mm	90 mm
Flexural and tensile stiffness of the composite structure, in KN/mm	16.126	18.000	23.530	25.000
Failure load, in KN	12.0	13.5	16.5	18

Under the simulated action of a negative bending moment, the bearing capacity of the pavement structure was much lower than that under the simulated action of a positive bending moment; the thickness of the pavement layer also had a large impact on the stiffness of the pavement structure; that is, the former is directly proportional to the latter.

The calculation results revealed that for single-layer pavement, when the pavement thickness was increased from 70 to 80 mm, an evident increase was observed in both the average flexural and tensile stiffness and average failure load of the composite pavement structure. When the load reached the peak, the bottom tensile material (high-content hybrid fibre polymer concrete) could prevent the expansion of cracking. Hence, all selected thicknesses of the pavement structures exhibited suitable tensile strength.

When the pavement structures were loaded under the simulated condition of a negative bending moment, cracks appeared at the bottom tensile surfaces of the structures. With a gradual increase in load, the bottom cracks gradually expanded upwards; when the cracks expanded to a certain height, the load was no longer increased. At this time, the cracks on the member expanded continuously, without sudden fracturing.

### Static test on double-layer pavement structure

In view of the driving comfort, permeability, and noise-reduction function of steel bridge deck pavement, this paper proposes a double-layer pavement structure (a pavement layer of high-content hybrid fibre polymer concrete and a wear layer of polymer lattice concrete). The wear layer has high permeability and performs the functions of improving the anti-sliding performance and distributing the loads of the vehicle wheels. The double-layer structure was paved from bottom to top: 12 mm steel plate + interface-bonding layer I + 70-mm-thick high-content hybrid fibre polymer concrete + interface-bonding layer II + 10 or 20-mm-thick polymer lattice concrete. Similar to the test conducted on a single-layer pavement structure, the test pieces were loaded under the action of a positive/negative bending moment.

The mechanical performance test conducted on the double-layer pavement structure mainly includes drawing the mid-span load–displacement curve, analysing the load–displacement curve in the linear elastic stage, calculating the flexural and tensile stiffness of the structure and the contribution of the wear layer to the flexural and tensile stiffness of the structure, and analysing the failure load on the structure and the form of the structure with failure load.

#### Performance analysis of the pavement structure under the simulated action of positive bending moment

**Figure 7:**
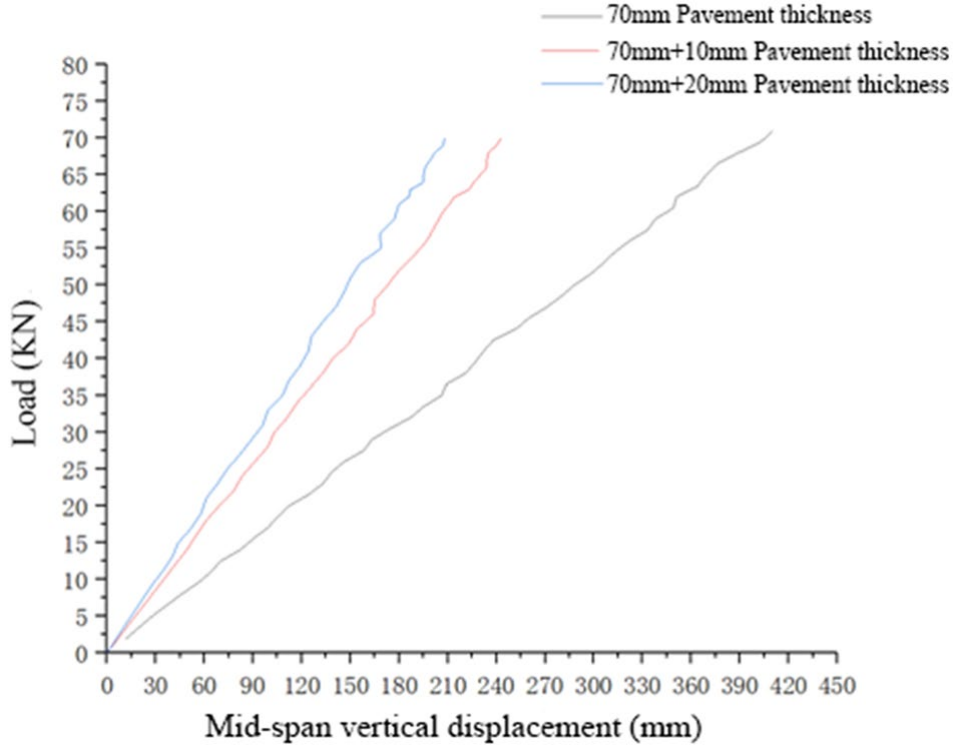
Load–displacement curve of a double-layer pavement structure with different pavement thicknesses in the linear elastic stage under the simulated action of a positive bending moment.

The contribution of the wear layer to the flexural and tensile stiffness of the pavement structure was calculated by analysing the load–displacement curve of the double-layer pavement structure and comparing it with that of a 70-mm-thick single-layer pavement structure. The following figure shows the mid-span load–displacement curve in the linear elastic stage. The flexural and tensile stiffness and failure load of the composite structure formed by different thicknesses of double-layer pavement under the action of a positive bending moment are presented in Table 5 and Fig. 7.

**Table 5:** Flexural and tensile stiffness and failure load of the composite structure formed by different thicknesses of double-layer pavement under the action of a positive bending moment.

Pavement thickness	70 mm	70 mm + 10 mm	70 mm + 20 mm
Flexural and tensile stiffness of the composite structure, in KN/mm	17.445	28.740	33.478
Failure load, in KN	71	73	77

As revealed from the calculation results, there was a large difference between the load–displacement curve of the double-layer pavement structure in the linear elastic stage and that of the 70-mm-thick single-layer pavement structure. In detail, the flexural and tensile stiffness of the former structure (70 mm + 10 mm in thickness) was improved by 64.7% from that of the latter structure. When the wear layer thickness was increased from 10 to 20 mm, the flexural and tensile stiffness of the pavement structure was improved by only 16%, a variation that was not evident.

Regarding the failure load, there was no significant difference between the double-layer pavement structure and the 70-mm-thick single-layer pavement structure. The flexural and tensile stiffness and failure loads of 80- and 90-mm-thick single-layer pavement structures were both slightly higher than those of the ‘70 mm + 10 mm’ thick and ‘80 mm + 10 mm’ thick composite pavement structures. The main reason for this is that the single-layer pavement structure is well integrated and thus contributes significantly to the entire flexural and tensile stiffness and bearing capacity of the structure.

Under the simulated loading of a positive bending moment, the wear layer paved by polymer lattice concrete and the lower layer paved by high-content hybrid fibre polymer concrete jointly carried the forces on the structure. Similar to the single-layer pavement structure, cracks also appeared near the support as the load increased, with the main crack obliquely pointing to the loading point. When the load reached the peak, the cracks expanded deeply. However, as the two layers of the double-layer pavement structure were paved by different paving materials, the cracks did not reach the polymer lattice concrete of the wear layer, and the two layers were well bonded. Before displacement failure occurred on the two layers, no clear crack was observed on the wear layer, but partial concrete of the wear layer was crushed.

#### Performance analysis of the pavement structure under the simulated action of a negative bending moment

**Figure 8:**
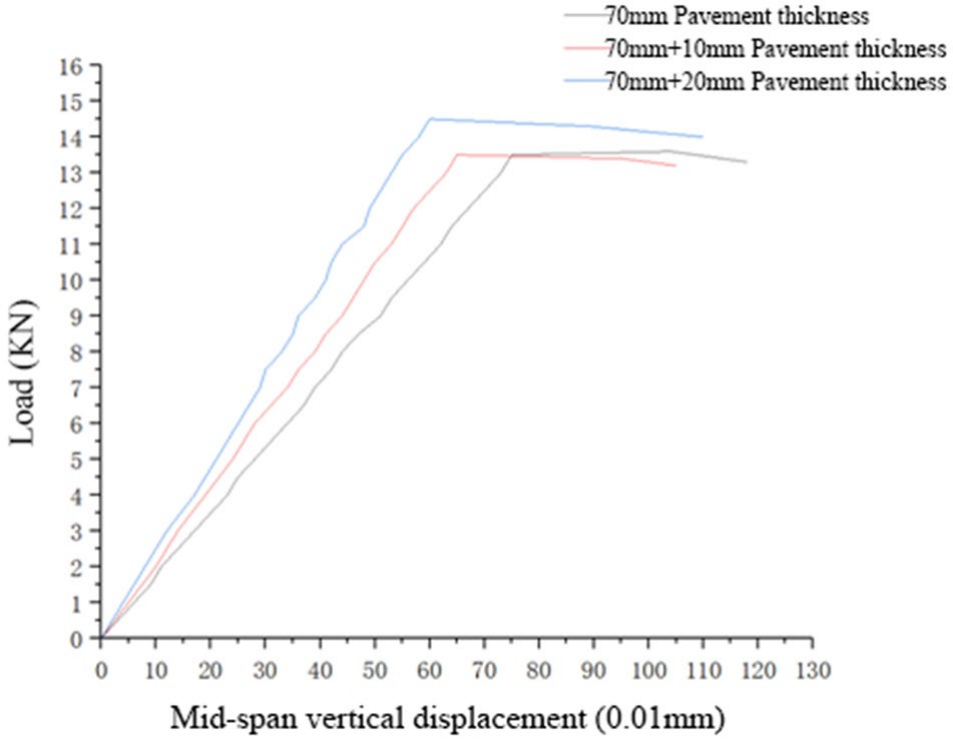
Load–displacement curves of double-layer pavement structures with different pavement thicknesses under the simulated action of a negative bending moment.

Under the simulated action of a positive/negative bending moment, the pavement structure showed a huge difference in bearing capacity, specifically in the load–displacement curve. Under the simulated action of a negative bending moment, the load–displacement curve of the double-layer pavement structure is vital for analysing the contribution of the wear layer to the flexural and tensile stiffness of the entire structure and the contribution of the thickness change in the wear layer to the entire flexural and tensile stiffness. The flexural and tensile stiffness and failure load of the composite structure formed by different thicknesses of the double-layer pavement structure under the action of a negative bending moment are displayed in Fig. 8 and Table 6.

**Table 6:** Flexural and tensile stiffness and failure load of the composite structure formed by different thicknesses of double-layer pavement under the action of a negative bending moment.

Pavement thickness	70 mm	70 mm + 10 mm	70 mm + 20 mm
Flexural and tensile stiffness of the composite structure, in KN/mm)	18.000	20.769	24.167
Failure load, in KN	13.5	13.5	14.5

The calculation results showed no evident difference between the load–displacement curve of the double-layer pavement structure under the action of a negative bending moment and that of the single-layer pavement structure. The flexural and tensile stiffness and failure load of the composite structure did not increase significantly with the thickness of the wear layer. Compared with the double-layer pavement structure, the single-layer pavement structure underwent a clear yield point elongation after the load reached the peak.

For the composite structure under the simulated action of a negative bending moment, cracks appeared on the top of a small beam test piece with an increase in load and gradually expanded from the wear layer paved by the polymer lattice concrete to the lower layer paved by the high-content hybrid fibre polymer concrete; after the cracks expanded to a certain height, the load reached the peak, and the displacement increased continuously at evidently increased acceleration. In the presence of cracks in the lower layer, the pavement structure could continuously bear a higher range of load until the cracks expanded to the critical value, the test piece could no longer bear a higher load, and failure occurred on the test piece.

## Conclusion

In this study, the structural test research method of indoor design was used to study the mechanical properties of the high-content hybrid fibre polymer concrete pavement structure. The two types of pavement structures and the composite pavement structures with different pavement thicknesses were subjected to small beam tests and loaded with positive and negative bending moments. Hence, the load–displacement curves and failure forms of the structures under the actions of positive and negative bending moments were obtained and used as the basis for analysing the stiffness of the composite pavement structures and verifying the anti-cracking enhancement mechanism of the two types of paving materials. The following small beam test results were obtained:

1. For a single-layer pavement structure, the 80-mm-thick pavement formed a composite structure, which had evidently higher flexural and tensile stiffness than the 70-mm-thick pavement structure. The ultimate bearing capacity of the pavement structures under the action of a negative bending moment is far lower than that under the action of a positive bending moment, and the variation in pavement thickness contributes little to the flexural and tensile stiffness and bearing capacity of the composite structures.
2. For the double-layer pavement structure, the composite structure formed by the lower layer of 70 mm-thick high content hybrid fibre polymer concrete and the upper wear layer of polymer lattice concrete significantly enhanced the flexural and tensile stiffness under the action of a positive bending moment, but no evident change was observed in the ultimate bearing capacity of the structure.
3. When the wear layer of the double-layer pavement structure was subjected to failure under load, the wear layer remained unbroken, and the upper layer remained well bonded with the lower layer. This verifies that this structure exhibits the superiority of ‘lattice + node + void’ and superior mechanical properties of bonding.
4. Regardless of the simulated action of a positive or negative bending moment, the pavement layer still exhibits certain ductile failure characteristics when the structure bears the ultimate load, which proves that high-content hybrid fibre polymer concrete exhibits suitable mechanical properties and is an ideal material for paving the steel bridge deck.

In short, the structure paved by high-content hybrid fibre polymer concrete exhibits suitable structural performance. This material has good application prospects. Future research should further emphasise the durability of the pavement structure.

## Data Availability

All data generated or analyzed during this study are included in this published article.

## Abbreviations

GA: Grouted asphalt
SMA: Stone mastic asphalt
UHPC: Ultrahigh-performance concrete
